# Anterior Mediastinal Lesion Segmentation Based on Two-Stage 3D ResUNet With Attention Gates and Lung Segmentation

**DOI:** 10.3389/fonc.2020.618357

**Published:** 2021-02-08

**Authors:** Su Huang, Xiaowei Han, Jingfan Fan, Jing Chen, Lei Du, Wenwen Gao, Bing Liu, Yue Chen, Xiuxiu Liu, Yige Wang, Danni Ai, Guolin Ma, Jian Yang

**Affiliations:** ^1^ Beijing Engineering Research Center of Mixed Reality and Advanced Display, School of Optics and Photonics, Beijing Institute of Technology, Beijing, China; ^2^ Department of Radiology, The Affiliated Drum Tower Hospital of Nanjing University Medical School, Nanjing, China; ^3^ Department of Radiology, China-Japan Friendship Hospital, Beijing, China

**Keywords:** anterior mediastinal lesion segmentation, deep learning, two-stage 3D ResUNet, attention gates, lung segmentation model

## Abstract

**Objectives:**

Anterior mediastinal disease is a common disease in the chest. Computed tomography (CT), as an important imaging technology, is widely used in the diagnosis of mediastinal diseases. Doctors find it difficult to distinguish lesions in CT images because of image artifact, intensity inhomogeneity, and their similarity with other tissues. Direct segmentation of lesions can provide doctors a method to better subtract the features of the lesions, thereby improving the accuracy of diagnosis.

**Method:**

As the trend of image processing technology, deep learning is more accurate in image segmentation than traditional methods. We employ a two-stage 3D ResUNet network combined with lung segmentation to segment CT images. Given that the mediastinum is between the two lungs, the original image is clipped through the lung mask to remove some noises that may affect the segmentation of the lesion. To capture the feature of the lesions, we design a two-stage network structure. In the first stage, the features of the lesion are learned from the low-resolution downsampled image, and the segmentation results under a rough scale are obtained. The results are concatenated with the original image and encoded into the second stage to capture more accurate segmentation information from the image. In addition, attention gates are introduced in the upsampling of the network, and these gates can focus on the lesion and play a role in filtering the features. The proposed method has achieved good results in the segmentation of the anterior mediastinal.

**Results:**

The proposed method was verified on 230 patients, and the anterior mediastinal lesions were well segmented. The average Dice coefficient reached 87.73%. Compared with the model without lung segmentation, the model with lung segmentation greatly improved the accuracy of lesion segmentation by approximately 9%. The addition of attention gates slightly improved the segmentation accuracy.

**Conclusion:**

The proposed automatic segmentation method has achieved good results in clinical data. In clinical application, automatic segmentation of lesions can assist doctors in the diagnosis of diseases and may facilitate the automated diagnosis of illnesses in the future.

## Introduction

Given the high incidence of chest diseases, the anterior mediastinal disease is an urgent medical condition. This disease includes a wide variety of illnesses (1–4), the most common of which are thymoma (5, 6), lymphoma, and more than 10 other illnesses (7, 8). However, despite its physiological significance, anterior mediastinal disease has received little attention in medical image analysis. On the one hand, the chest images used for detection often contain information irrelevant to the lesion. On the other hand, the anterior mediastinal lesion is characterized by low contrast, irregular shape, different sizes, and unstable anatomical positions. These features lead to challenges to the image acquisition and analysis of anterior mediastinal disease and difficulties for doctors to make diagnosis. Image segmentation can characterize the size and delineate the boundary of the lesion (9); thus, this process can assist doctors in diagnosing the disease. In radiomics, segmentation of the lesion area is usually the first step for automatic diagnosis (10–13). As a part of traditional radiomics, segmentation is usually performed by using traditional feature engineering methods. This handcrafted feature has certain limitations. Some traditional segmentation methods also need manual interaction, such as region growth (14) and graphcut (15, 16). Some conventional techniques, such as snakes (17, 18) and active contour model (19, 20), require the manual setting of many parameters. These processes cannot achieve fully automatic results, and the segmentation results for low-contrast medical images are poor.

Segmentation is an essential prerequisite in medical image analysis for image-guided intervention (21), radiotherapy (22), or improved radiological diagnostics. With the rapid development of segmentation technology, deep learning has become one of the mainstream technologies. Li et al. (23) combined the features extracted with CNN (convolutional neural networks) and those extracted with radiomics to predict the ICH1 in low-level neural mutations in gliomas. Fully convolutional networks (24), U-Net (25), and VNet (26) are commonly used architectures. Despite their good representation in feature description, these architectures rely on CNNs when the target lesions show large inter-patient variation in terms of shape and size. These frameworks have been applied in many areas, including abdominal computed tomography (CT) segmentation (27), lung CT nodule detection (28), and liver segmentation (29). However, these approaches lead to the excessive use of computational resources and model parameters. Compared with organ segmentation and some kind of tumor segmentation, anterior mediastinal lesions have various shapes and variable size. Nevertheless, no thymus-specific segmentation algorithm that uses deep learning has been proposed because of the lack of data and high difficulty of annotation.

In this study, to improve the segmentation accuracy, lesions of the anterior mediastinum have been segmented automatically by using two-stage Res3DUNet. Based on the 3DUnet network, the automatic segmentation of lung model is added as the data preprocessing stage, and image clipping is used to remove the information irrelevant to the lesion and reduce noise in the image. During network construction, the attention mechanism is added, such that the network is focused more on the areas of interest. These improvements result in good segmentation.

The contribution of this report includes the proposal of a two-stage Res3DUnet network structure to automatically segment the lesion area of the anterior mediastinal disease. Thus, a reference is provided for doctors to facilitate the diagnosis of the disease. In addition, information on the anatomic location of the lung is used to remove the irrelevant part of the image and relatively enlarge the region of interest. Attention gates (AGs) are added to the network to improve the accuracy of the model.

## Materials and Methods

### The Whole Framework

**Figure 1:**
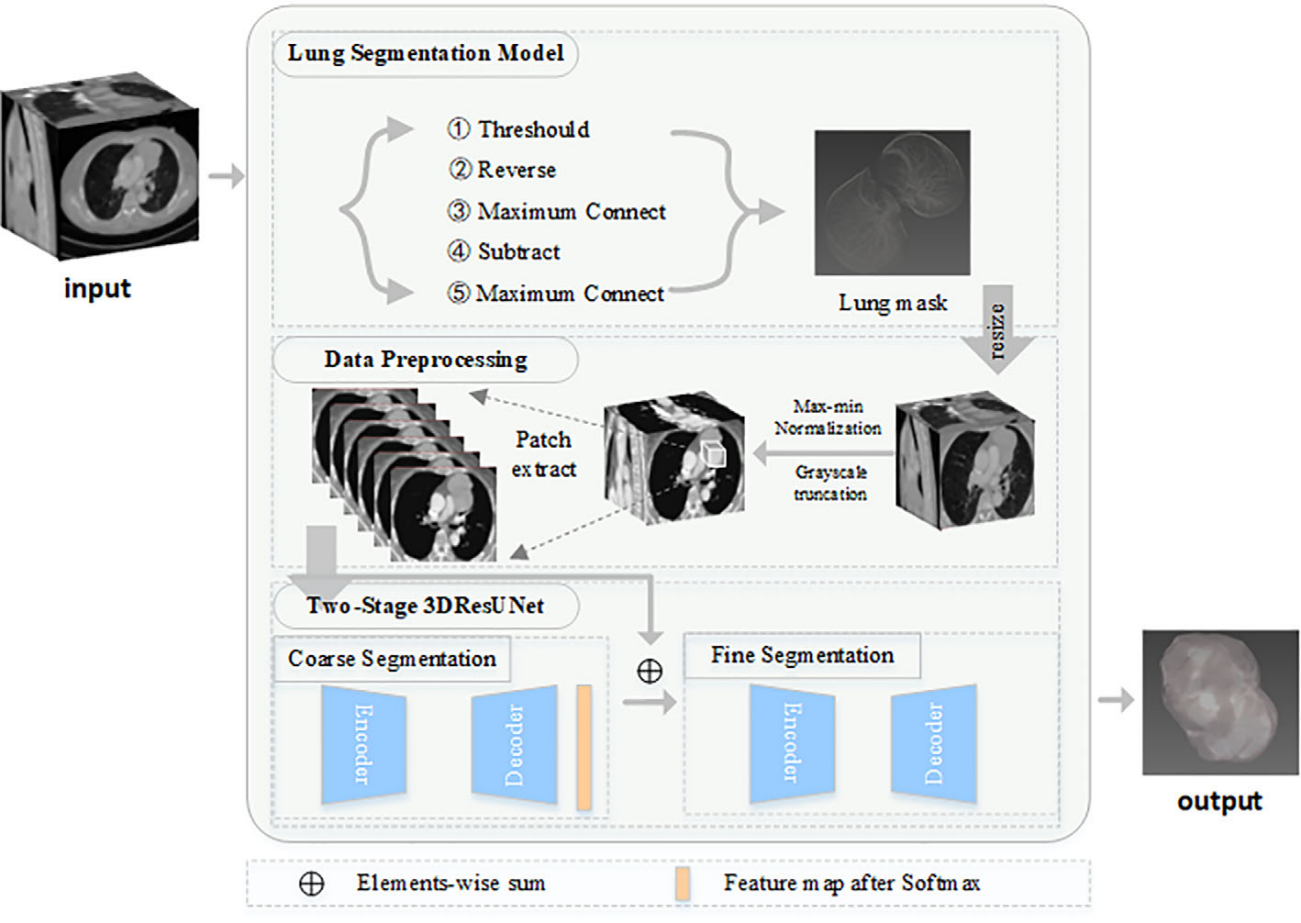
Framework of proposed model.

Given that the lesions differ in shape and size, inspired by Refs. (30) and (31), the lesions are segmented from coarse to fine by using a two-stage network structure (**Figure 1**). The first stage is mainly performed to determine the specific location of the lesion. The second stage is conducted for the fine segmentation of the lesion. The input of the second stage is concatenated the feature maps, which is out from the first stage with the high-resolution image. The parameter settings of the two-stage network are consistent, and cross-entropy (25) loss is used for end-to-end optimization and training.

### Lung Segmentation Model

Given that the anatomical structure of the thymus is roughly between the two lungs, the original image is clipped and preprocessed by the lung mask to remove some factors, such as the background plate, that may affect the segmentation of the lesion. Contrary to the conventional rotation and pixel truncation, the relative anatomical position of the lung and the anterior mediastinal lesion is used to clip the image. Specific pulmonary segmentation includes the following steps. To generate the threshold image, CT value lower than −300 is set as 0, and the CT value greater than −300 is set as 1. After the reverse operation of the threshold image, the maximum connected domain algorithm is used to obtain the thoracic cavity. The thoracic cavity is subtracted from the threshold image, and then the maximum connected domain is processed to remove a small amount of noise. Finally, the lung mask is obtained.

### Data Pre-progressing

The original image is clipped according to the lung mask, and the size of the image is resized to 256×256. Then, some irrelevant pixels are filtered by grayscale truncation, and the images are normalized by using the max-min normalization method.

### The Backbone of Network

**Figure 2:**
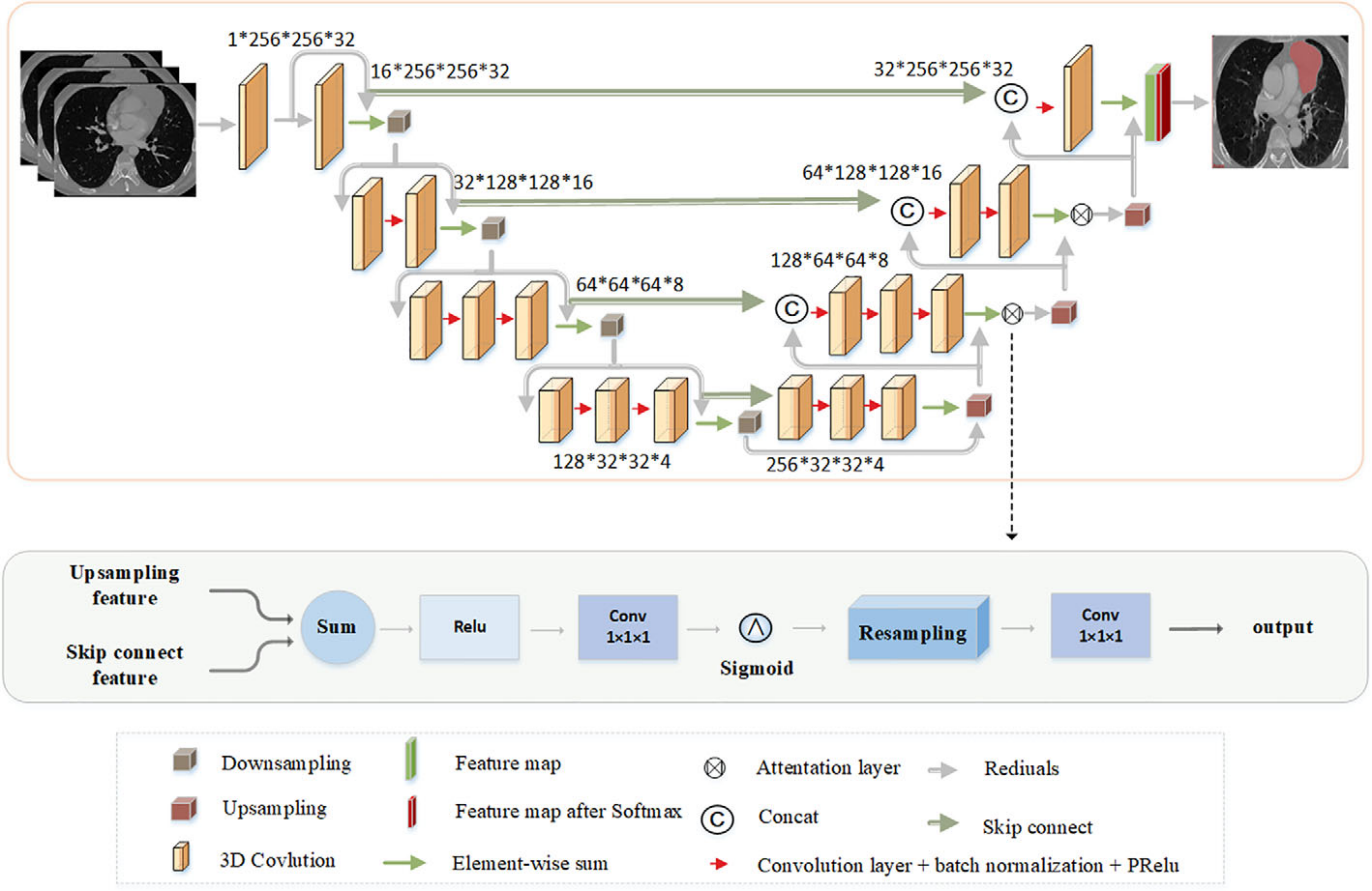
The architecture of network.

The network structure is shown in **Figure 2**. In this study, the kernel size of all convolutional layers is 3×3×3. The parameter 1×256×256×32 in turn represents channel, the height, the width, and the depth of the image. The network learns the residual function from the input and output at each stage. To prevent the disappearance of the gradient, the residual block is added in this report. After each downsampling, the height, width, and depth of the feature map become half of the former input. After each upsampling, the height, width, and depth of the feature map become twice of the former input. The convolution kernel size used by the last convolutional layer is 1×1×1, which keeps the size of the output image consistent with that of the original input image. Therefore, the semantic segmentation results can be obtained as the original input image size. According to Refs. (32) and (33), the attention mechanism between the corresponding downsampling and upsampling layer is added, and the attention mechanism can select features useful for lesion segmentation. Finally, Softmax is used to generate the segmentation probability graph of the lesions and background. To train the two-stage Res3DUNet, the cross-entropy loss is used to measure the difference between the prediction and ground-truth distributions by calculating the “gap” between the two distributions pixel by pixel. The cross-entropy formula is defined as follows:

(1) $$E=-\sum_{k\in \Omega }w_{k}(x)\log(p_{k(x)}(x))$$

where Ω represents the image; *k*(*x*) represents pixel *x*, which belongs to the *k_th_* class; and *w_k_*(*x*) denotes the weight of pixel *x* belonging to the *k*_th_ class.

### Attention Gates

The AGs can suppress the irrelevant and noisy responses of background areas without cropping the region of interest and training a large number of additional parameters in the network. To obtain a sufficiently large reception field to obtain the information in the semantic context, the feature map grid will usually use the downsampling strategy. However, with respect to small objects with large morphological changes, the CNN structure is difficult to reduce the false-positive error of prediction. To avoid these errors as much as possible, the proposed approach introduces AGs as reported in Refs. (32) and (33). AGs are used after the downsampling and upsampling features are integrated. The output of the AGs is the element-wise multiplication of the input feature-maps and attention coefficients, as follows:

(2) $$\overset{\wedge }{x_{i,c}}=x_{i,c}^{l}\cdot \alpha_{i,c}^{l}$$

where $x_{i,c}^{l}$ is the feature map from upper layer and $\alpha_{i,c}^{l}$ is the attention coefficient belonging to [0,1], which identifies the salient image regions and prunes feature responses to preserve only the activations relevant to a specific task.

## Results

### Data Preparation and Parameter Setting

The Institutional Ethics Review Committee of the China-Japan Friendship Hospital approved this retrospective study. A total of 230 cases were used in this experiment, including 116 cases from the China-Japan Friendship Hospital and 114 cases from the Nanjing Eastern Theater General Hospital. The CT images from the China-Japan Friendship Hospital were obtained with a variety of scanners, including a 16-row multi-detector CT (MDCT) (Toshiba Aquilion, Japan), 320-row MDCT (Toshiba Aquilion TM ONE, Japan), and a 256-row MDCT (GE revolution, USA). The CT images from the Nanjing Eastern Theater General Hospital were obtained with a 128-row MDCT (SIEMENS SMOATOM Definition, Germany). Two physicians with clinical experience outlined the ground-truth of the data. A total of 116 cases were used as the training data and 114 cases as the test data to distinguish the differences in the data caused by the imaging equipment and to better highlight the advantages of deep learning models. The settings in all experiments were consistent for all compared methods to ensure a fair comparison. In the experiments, “Adam” was chosen as the optimizer to optimize the target of the model, the training epoch was 300, and the learning rate was 0.0001. The evaluation metric use the Dice coefficient to describe the global segmentation performance with the ground-truth mask as Equation (3). The network was trained on one piece Nvidia 2080Ti (11 GB) GPU machine. The network was implemented using the PyTorch framework.

(3) $$D=\frac{2\sum_{i}^{N}p_{i}g_{i}}{\sum_{i}^{N}p_{i}^{2}+\sum_{i}^{N}g_{i}^{2}}$$

where *p_i_* is the i-th pixel value of the predicted image and *g_i_* is the i-th pixel value in the ground truth.

### Result of Adding Lung Segmentation

**Figure 3:**
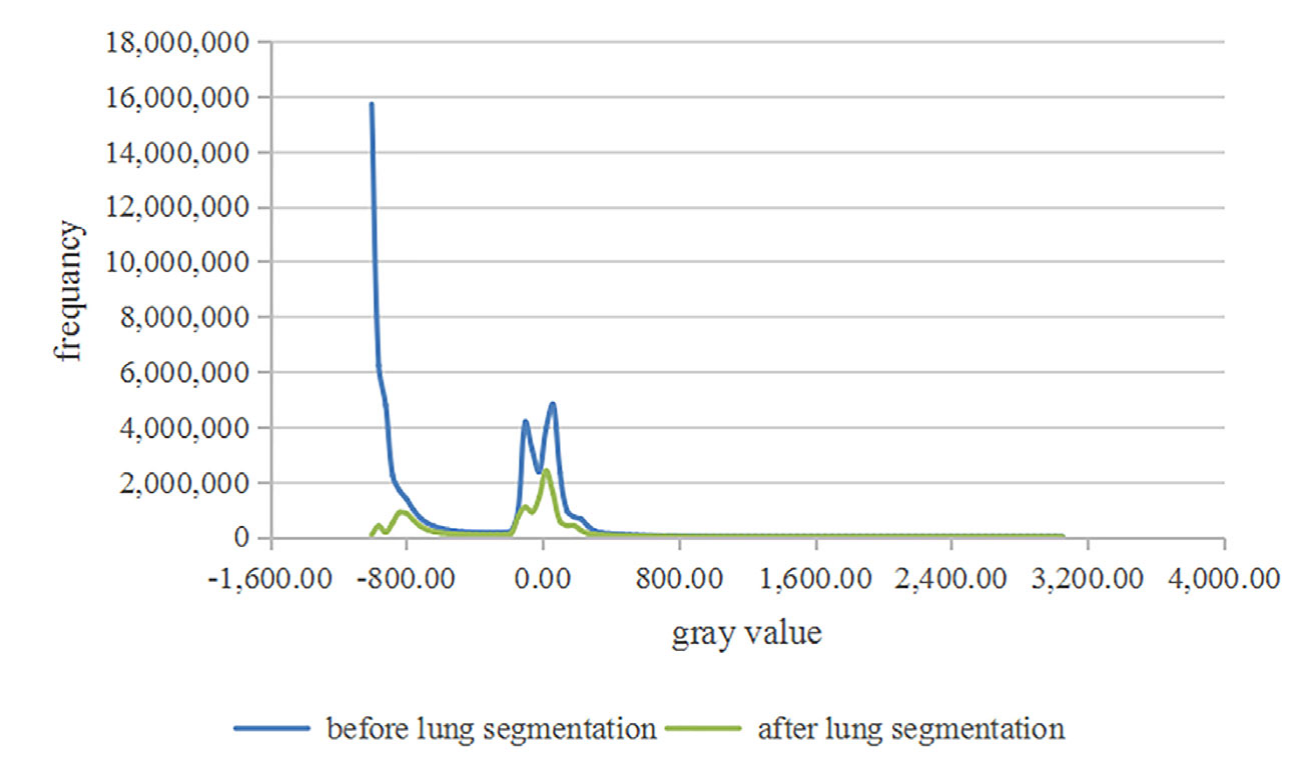
The distribution of gray values.

For the CT images, all images are grayscale corpuscles. In common segmentation tasks, noise is easy to be introduced, which reduces the accuracy of segmentation. Lung segmentation can separate the lung from the whole CT image, reduce the interference of background template, and enhance the segmentation results. The distribution of gray values during lung segmentation is contracted (**Figure 3**) because we only keep the area covered by the lung mask. After the image is cut according to the anatomical position of the lung, the noise is reduced considerably. To verify the effectiveness of the lung segmentation, the experimental results are compared with or without the lung segmentation, and the Dice coefficient is calculated for the segmentation results. The results in **Table 1** show that adding the lung segmentation can improve the accuracy of the segmentation results.

**Table 1:** Results of whether adding lung segmentation model.

Method	Dice (%)
Two-stage 3D ResUNet	78.65
Two-stage 3D ResUNet+lung segmentation	85.43

### Result of Different Models

In the segmentation model, AGs are introduced to highlight the segmentation region and its important features. Therefore, based on the lung segmentation, the effects of the attention mechanism on the segmentation task are compared. The results are shown in **Table 2**. Introduction of the attention mechanism improves the accuracy of the results. Then, the results of the nnUNet (34) to the present results are compared. **Table 2** shows the performance of different segmentation models. The two-stage 3D ResUNet with attention gates have better performance in terms of comprehensive time efficiency and Dice coefficient.

**Table 2:** Performance of different models.

Method	Dice (%)	Training Time(s)
nnUnet (34)	87.78	289
Two-stage 3D ResUNet+lung segmentation	85.43	40
Two-stage 3D ResUNet +lung segmentation+ Attention	87.73	44

### Results of Visualization

**Figure 4:**
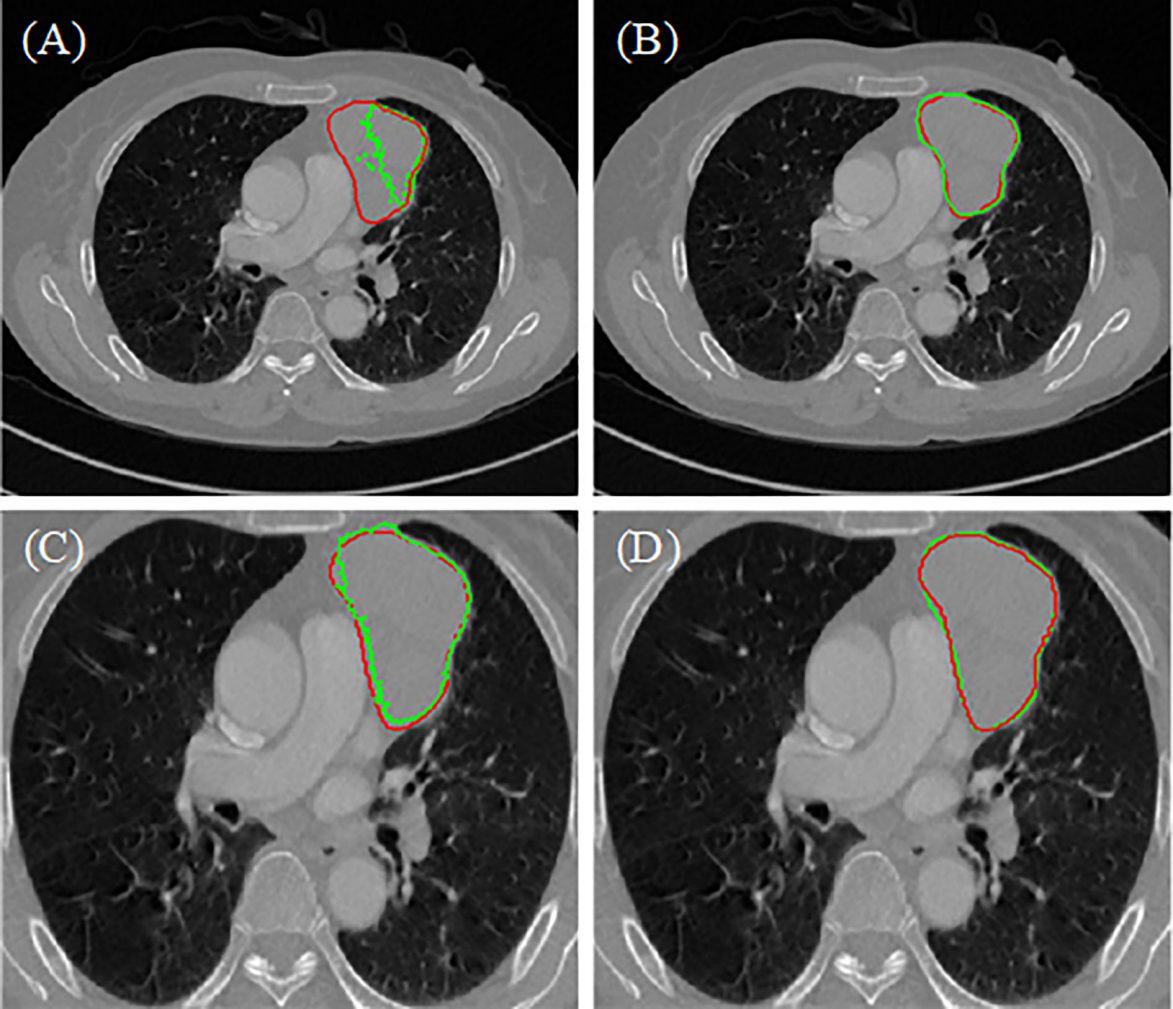
Results of different model **(A)** two-stage 3D ResUNet, **(B)** nnUnet, **(C)** two-stage 3D ResUNet+lung segmentation, **(D)** two-stage 3D ResUNet + lung segmentation + Attention.

The segmentation results of the different methods in two hospitals are illustrated in **Figure 4**. The predicted areas and ground-truth annotations are shown with green and red, respectively. Among all the compared segmentation methods, the nnUnet provides better dice coefficient but is time consuming. The proposed model provides similar results but consumes less time. Compared to no lung segmentation model, the ROI of the lung segmentation model is more bigger, so that when doing convolutional operation, the receptive field will be bigger relatively. Because the attention mechanism helps to focus on the area of interest, the lesion predicted by the proposed model is clearly closer to the ground truth annotation. Therefore, the proposed model has more potential on the segmentation of anterior mediastinal lesions than the state-of-the-art method.

## Discussion

This study shows that deep learning can achieve good results in the segmentation of anterior mediastinal lesions and provides a relatively reliable basis for subsequent clinical diagnosis. Full automatic segmentation can greatly improve the work efficiency of doctors.

The anterior mediastinal lesion is characterized by low contrast, uneven gray distribution, and close gray level of surrounding tissues. Thus, assessing the area of the lesion by the naked eye is difficult. With the improvement of the computing power, deep learning is widely used in tumor segmentation, organ separation, and pathological image segmentation. However, very little work on the segmentation of anterior mediastinal lesions has been performed. Existing work is mostly for the screening and diagnosis of mediastinal disease. As the first step of disease diagnosis and evaluation, lesion segmentation is usually outlined manually by an experienced doctor. He et al. (10) applied the RadCloud platform (Huiying Medical Technology Co., Ltd., Beijing, China) to delineate anterior mediastinal lesions. An experienced doctor confirmed the boundary of the lesion and then used machine learning to extract the characteristics of the lesion. Zhu et al. (35) used ITK-SNAP to manually segment thymoma to prepare for the extraction of lesion features by radiomics and deep learning methods. The two-stage 3DResUNet with attention based on the lung segmentation model proposed in this paper can automatically segment the lesions and still achieve relatively good results when the sources of the training set and the test set are different. This result indicates that the model has a certain degree of robustness. The lung segmentation model uses the anatomical position of the lungs to crop the image and removes irrelevant information brought in when CT images are collected. This process effectively reduces the noise interference and prevents the network from learning useless information. Thus, the accuracy and efficiency of the model are improved. The classic nnUnet is also compared with the proposed model. The result of the proposed method is close to that from nnUnet (34), but the former method consumed less time. Thus, the effectiveness of the proposed model is verified.

This study still has some limitations. On the one hand, the scale of the data set will limit the performance of the model. When the network learns the features of data, the network will be affected by the amount of data. This phenomenon results in learned features that are not comprehensive enough, and the generalization ability of the model has not been fully verified. On the other hand, the result of segmentation has inaccurate boundaries. Given that the pixel value of the lesion area is close to that of the surrounding tissues, the precise boundary is difficult to obtain. Xu et al. (36) added the traditional active contour model as a loss function to deep learning to segment the left ventricle, playing a role in boundary constraints.

Further research will include the combination of traditional segmentation methods and deep learning techniques, because the former methods have good interpretability. Such characteristics can strengthen the constraints on the boundary and shape of the lesion and obtain more accurate segmentation results. In addition, the application of automatic segmentation in disease screening, disease risk assessment, and other aspects can be explored to provide more intelligent and comprehensive support for clinical use.

## Conclusion

In this study, the two-stage Res3DUnet is applied to fully automate the segmentation of the anterior mediastinal lesions from ordinary CT images. The two-stage Res3DUnet combined lung segmentation model and attention mechanism can enhance the accuracy of the result. The two-stage Res3DUnet segments the lesion from coarse to fine. The lung segmentation model can not only crop the unrelated background information but also enlarge the receptive field in the lesion. The attention mechanism focuses on the ROI without extra spatial consumption. The proposed approach is evaluated using the datasets collected from two different hospitals. The experimental results show that deep learning has great potential in the segmentation of anterior mediastinal lesions. The two-stage network architecture is more advantageous than the classical network architecture and is suitable for the segmentation of medical images.

## Data Availability Statement

The original contributions presented in the study are included in the article/**Supplementary Materials**. Further inquiries can be directed to the corresponding authors.

## Ethics Statement

The Institutional Ethics Review Committee of the China-Japan Friendship Hospital approved this retrospective study.

## Author Contributions

SH performed the deep learning model, analyzed the data, and drafted the manuscript. XH acquired, labeled the data, and drafted the manuscript. JF analyzed the deep learning model. JC contributed to revisions and corrections. LD, WG, and BL analyzed and explained the imaging data. YC, XL, and YW acquired the clinical information and revised the manuscript. DA, GM, and JY designed the study and revised the manuscript. All authors contributed to the article and approved the submitted version.

## Funding

This work was supported by the National Key R&D Program of China (2019YFC0119300), and the National Science Foundation Program of China (61971040, 81971585, 91959123, 81627803, 62071048, and 61771056).

## Conflict of Interest

The authors declare that the research was conducted in the absence of any commercial or financial relationships that could be construed as a potential conflict of interest.
